# 

**Published:** 1855

**Authors:** 


EDITED BY

BENJAMIN W. RICHARDSON, M.D.,

PHYSICIAN TO THE ROYAL INFIRMARY FOR DISEASES OF THE CHEST.

S.G.O.
INDEXED

NATIONAL HEALTH  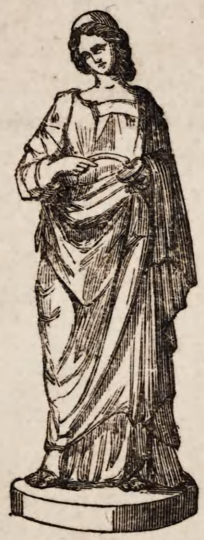 IS NATIONAL WEALTH.

Surgeon Genl's Office
6213
LIBRARY.
Washington D.C.

'ΥΓΙΕΙΑ

*πρεσβίστη μακάρων.*

VOL. I.

no. 1-4

PUBLISHED FOR THE PROPRIETOR, BY

THOMAS RICHARDS, 37, GREAT QUEEN STREET.

1855.

WI
J0859m

LONDON:
T. RICHARDS, 37, GREAT QUEEN STREET.

### INDEX.

Acids, Vegetable and Mineral, in Cholera, *rev.*, 154
Adulterants, dictionary of, 238
Adulteration of food, report of committee on, 319
Army, British, Sanitary Regulations of, *rev.*, 32; report of, 69; sanitary concerns of, Dr. Davy on, 210
—— French in the East, health of, 88
—— Xenophon on the sanitary management of an, 70
Atmosphere in relation to disease, Mr. Hingeston on, 357
Banbury, Water Supply of, *rev.*, 271
Barracks and hospitals, construction and arrangements of, 99
Bedford, sanitary report of, Dr. Barker on, 67
Berlin, epidemics in, 52, 168; cholera in, 273
Births, deaths and marriages in United States, 189
——— and deaths in St. Petersburg and Moscow, 426
Bombay, mortality of, 180
Bread, lime-water in formation of, 78
Budd, Dr. G., Diseases of Stomach, *rev.*, 395
Burial regulations in England, 312
Burials in charcoal, 89, 207
Charcoal applied to Sanitary Purposes, *rev.*, 46; to burial purposes, 89, 207; manufacture and cost of, Mr. Durden on, 213
Chicory, returns regarding, 319
Cholera, Transfusion of Milk in, *rev.*, 154; use of vegetable and mineral acids in, *ib.*; diffusion of in Scotland, 173; epidemic in Berlin, 273; report of Committee of Board of Health, 318, 397; in St. James's parish, 396; in relation to atmospheric changes, 396; propagation of, 402; in Jamaica, 415
Climate, Weather, and Disease, Mr. Haviland on, *rev.*, 393
Colliery District in Staffordshire, *rev.*, 271
Cork, extramural interment in, Dr. Pickells on, 223
Coroners' inquests in England and Wales, 194
Cranium, Human, Mr. Dunn on, *rev.*, 270
Dead bodies, exposure of, in Spain, 320
Dictionary of foods and drinks, 121, 238, 370
Drainage, metropolitan, 82
Dudley, Sanitary State of, Mr. Houghton on, *rev.*, 272
Dust, return, in model lodging-houses, 75
Earth-boring machinery, 185
Eating and drinking, rules for, 395
Epidemics, Chronological Survey of, *rev.*, 41; in London, 51, 156, 400; of England, 155; of Ireland, 171; in Berlin, 52, 168
Epidemic and endemic diseases, local reports of, 55, 158, 299, 403
Epidemiological Society, objects of, 3
Epizootic diseases, report on, 61
Eyes, trades which affect the, 72
Fireplace, smokeless, Dr. Arnott's, 267
Food, animal, diseased, 86
France, sickness and mortality in, 194
Fuel in its Sanitary Applications, *rev.*, 386
Gavin, Hector, M.D., sanitary labours of, 98
Glasgow, sanitary report of, 63
Graveyards in London, 82; statistics of, Dr. Webster on, 218, *See* Burials and Interments
Gymnastics, a Branch of Education, *rev.*, 48, 269
Harrogate Waters, *rev.*, 270
Hastings district, health and mortality of, Dr. Greenhill on, 416
Health, laws of, 79; necessity of education in, 5, 74, 92; Rev. Mr. Lyttelton on, 111; offences against, 85
———, disease and longevity, M. Flourens on, *rev.*, 149

Health, Board of, public bills and reports, 80, 109
Horseflesh as an article of food, 192
Hospitals and barracks, construction and arrangements of, 99
Hygiene, Personal and Domestic, *rev.*, 395. *See* Health
Hygienic jurisprudence, 80, 197, 317, 428
India, soldier life in, 187; obstacles to vaccination in, 195
Instinct, human, influence of in prevention and cure of disease, Mr. T. Hunt on, 103
Interments, extramural, 89, 223, 399
Introduction, 1
Islington, nuisances in, 311
Lambeth, sanitary condition of, 178
Landed proprietor, every man a, 236
London, epidemics in, 51, 156, 400
Longevity and Amount of Human Life on the globe, M. Flourens on, *rev.*, 152; examples of in Scotland, 329
Lime-water in making bread, 78
Madeira, Climate of, *rev.*, 270
Malt consumed in united kingdom, 314
Medical police of London, 324; officers of health, 324, 428
Meteorological Society, report of, *rev.*, 49; Association, Scottish, *rev.*, 272
Metropolis, local management Act, 316
Mortality, in relation to number of medical practitioners, 76; comparative, of manufacturing and agricultural districts, Dr. Black on, 364
Nuisances removal Act, 315
Oxford, Sanitary Report of, *rev.*, 153
Paint, Merton's sanitary, 185
Paris, sanitary state of, 310
Parliamentary acts, bills, and returns, 80, 197, 315, 430
Poor, sanitary and social condition of the, 8, 117
Potato, substitute for, 79
Railway travelling a cause of disease of the brain, 196, 422
Rape-cake, adulteration of, 86
Registration of facts by practitioners, 15
Reports on cities, towns, and districts, 63, 174, 309
———, local, of epidemic and endemic diseases, 55, 158, 299
Rome, Antient, Sanitary Regulations of, Mr. Haviland on, *rev.*, 141, 261, 379
Romsey, sanitary state of, 309
Russia, food resources of, 321; births and deaths in, 421
Sanitary and social science, 63, 174, 309, 416; legislation, 80, 197, 201, 315, 428; police, Dr. Druitt on, 15, 359; question, financial aspects of the, *rev.*, 268; questions, scientific investigation of, 29. *See* also Health
Scotland, longevity in, 329
Season, the, its diseases, and their prevention, 366
Sewage of towns applied to agriculture, 78
Sewers cleansed by steam jet, 77
Small-pox in Quebec, 113
Spirits, British, exported, 195; consumed in Scotland, 196; in arts and manufactures, 314
Swansea, sanitary report of, Mr. Michael on, 65, 153, 174
Trades, offensive, 267
Tropical climates, rules for preservation of health in, Dr. Daniell on, 22
United States, births, deaths, and marriages in, 189; mortality in, 426
Vaccination in India, obstacles to, 195
——— bill, 317
Ventilation, Dr. Chowne's system, 181; Duvoir's, 422; Dr. Cowan on, 423
Water, good in clay districts, 78; metropolitan supply of, 83, 420; Supply in Relation to Health and Disease, *rev.*, 130; purification of, 185; supply to Banbury, 271
——— filter, a household, 399
Wine-docks, London, sanitary taste of, 326
Workhouse, life in a, 204; accommodation in England, 313; returns, London, 314

